# A Galactose-Binding Lectin Isolated from *Aplysia kurodai* (Sea Hare) Eggs Inhibits Streptolysin-Induced Hemolysis

**DOI:** 10.3390/molecules190913990

**Published:** 2014-09-05

**Authors:** Imtiaj Hasan, Miharu Watanabe, Naoto Ishizaki, Yoshiko Sugita-Konishi, Yasushi Kawakami, Jun Suzuki, Chikaku Dogasaki, Sultana Rajia, Sarkar M. A. Kawsar, Yasuhiro Koide, Robert A. Kanaly, Shigeki Sugawara, Masahiro Hosono, Yukiko Ogawa, Yuki Fujii, Hideyuki Iriko, Jiharu Hamako, Taei Matsui, Yasuhiro Ozeki

**Affiliations:** 1 Laboratories of Glycobiology & Marine Biochemistry and Molecular Toxicology, Department of Life and Environmental System Science, Graduate School of NanoBio Sciences, Yokohama City University, 22-2 Seto, Kanazawa-ku, Yokohama 236-0027, Japan; E-Mails: hasanimtiaj@yahoo.co.uk (I.H.); rajia_bio@yahoo.com (S.R.); yasukoide04@yahoo.co.jp (Y.K.); kanaly@yokohama-cu.ac.jp (R.A.K.); 2 School of Life and Environmental Science, Azabu University, 1-17-71, Fuchinobe, Chuo-ku, Sagamihara, Kanagawa 252-5201, Japan; E-Mails: salty-soybean@kuh.biglobe.ne.jp (M.W.); ishizaki@azabu-u.ac.jp (N.I.); y-konishi@azabu-u.ac.jp (Y.S-K.); yasushi@azabu-u.ac.jp (Y.K.); suzukij@azabu-u.ac.jp (J.S.); dogasaki@azabu-u.ac.jp (C.D.); 3 Department of Biochemistry and Molecular Biology, Faculty of Science, University of Rajshahi, Rajshahi-6205, Bangladesh; 4 Department of Natural Science, Varendra University, Rajshahi-6204, Bangladesh; 5 Department of Chemistry, Faculty of Sciences, University of Chittagong, Chittagong-4331, Bangladesh; E-Mail: akawsarabe@yahoo.com; 6 Division of Cell Recognition Study, Institute of Molecular Biomembrane and Glycobiology, Tohoku Pharmaceutical University, 4-4-1 Komatsushima, Aoba-ku, Sendai 981-8558, Japan; E-Mails: ssuga@tohoku-pharm.ac.jp (S.S.); mhosono@tohoku-pharm.ac.jp (M.H.); 7 Department of Pharmacy, Faculty of Pharmaceutical Science, Nagasaki International University, 2825-7 Huis Ten Bosch, Sasebo, Nagasaki 859-3298, Japan; E-Mails: yogawa@niu.ac.jp (Y.O.); yfujii@niu.ac.jp (Y.F.); 8 Department of Parasitology, Graduate School of Health Sciences, Kobe University, 7-10-2, Tomogaoka, Suma-ku, Kobe 654-0142, Japan; E-Mail: iriko@koala.kobe-u.ac.jp; 9 Department of Biology, School of Health Sciences, Fujita Health University, Toyoake, Aichi 470-1192, Japan; E-Mails: jhamako@fujita-hu.ac.jp (J.H.); tmatsui@fujita-hu.ac.jp (T.M.)

**Keywords:** *Aplysia kurodai*, lectin, hemolysis, sea hare eggs, lectin, *Streptococcus pyogenes*, streptolysin

## Abstract

A specific galactose-binding lectin was shown to inhibit the hemolytic effect of streptolysin O (SLO), an exotoxin produced by *Streptococcus pyogenes*. Commercially available lectins that recognize *N*-acetyllactosamine (ECA), T-antigen (PNA), and Tn-antigen (ABA) agglutinated rabbit erythrocytes, but had no effect on SLO-induced hemolysis. In contrast, SLO-induced hemolysis was inhibited by AKL, a lectin purified from sea hare (*Aplysia kurodai*) eggs that recognizes α-galactoside oligosaccharides. This inhibitory effect was blocked by the co-presence of d-galactose, which binds to AKL. A possible explanation for these findings is that cholesterol-enriched microdomains containing glycosphingolipids in the erythrocyte membrane become occupied by tightly stacked lectin molecules, blocking the interaction between cholesterol and SLO that would otherwise result in penetration of the membrane. Growth of *S. pyogenes* was inhibited by lectins from a marine invertebrate (AKL) and a mushroom (ABA), but was promoted by a plant lectin (ECA). Both these inhibitory and promoting effects were blocked by co-presence of galactose in the culture medium. Our findings demonstrate the importance of glycans and lectins in regulating mechanisms of toxicity, creation of pores in the target cell membrane, and bacterial growth.

## 1. Introduction

Exotoxins are a type of harmful protein secreted by pathogenic microorganisms that damage the host through a specific infectious mechanism. Cholera toxin, a well-known exotoxin produced by the Gram-negative bacterium *Vibrio cholerae*, binds to ganglioside GM1 (NeuAcα2-3(Galβ1-3GalNAcβ1-4)Galβ1-4Glcβ1-Ceramide) on the target cell membrane to induce cAMP activation and consequent removal of chloride ion (Cl^−^) and water from cells, resulting in severe and potentially fatal diarrhea [1]. Verotoxin, an exotoxin secreted by *Escherichia coli* O157, binds to the glycosphingolipid (GSL) receptor Gb3 (Galα1-4Galβ1-4Glcβ1-Ceramide), resulting in blocked intracellular protein synthesis and consequent hemolysis and cell death [2].

Group A *Streptococcus pyogenes* (GAS) are Gram-positive bacteria found in human throat and skin. Streptolysin O (SLO) is an oxygen-labile, thiol-activated hemolytic exotoxin secreted by GAS [3]. It is a 63-kDa protein that may cause respiratory infection, glomerulonephritis, or multiple organ failure with hemolysis in the host [4]. In contrast to cholera toxin and verotoxin, which bind to GSL glycans, SLO binds to cholesterol molecules in the erythrocyte (red blood cell; RBC) membrane, leading to membrane perforation and hemolysis [5]. Pathogenic forms of GAS may cause food poisoning by secreting other virulence factors besides SLO; e.g., streptolysin S (oxygen-stable hemolytic peptide), *S. pyogenes* exotoxin B (cysteine protease), and NADase (nicotinamide adenine dinucleotide glycohydrolase) [6].

All animals have hemagglutinins or carbohydrate-binding proteins (lectins) that recognize specific glycan structures of glycoconjugates and are therefore able to agglutinate RBCs. Lectins are also involved in infectious, toxic, hemolytic, anti-microbial, anti-cancer, and cell signaling processes [7]. A variety of galactose (Gal)-binding lectins have been found in marine invertebrates that can distinguish between α- and β-anomers of sugars on the surface of vertebrate RBCs [8]. Since the hemagglutination activity induced by by lectins fuses erythrocyte membrane, we happened to have an idea to inhibit the hemolysis toxin by this protens. Our previous studies show that lectins purified from *Aplysia* (sea hare; phylum Mollusca, class Gastropoda) recognize α- and β-galactosides of GSLs and glycoproteins, and also display cytotoxic effects [9,10]. In analogy to GSL-specific lectins, there is a possibility that SLO exerts a hemolytic activity that targets the GSL-enriched microdomain (GEM) of RBCs.

Specific structures such as poly-*N*-acetyl-d-galactosamine, *N*-linked high mannose-type oligosaccharide, and O-linked oligosaccharide containing *N*,*N*'-diacetylbacillosamine have been identified in the peptidoglycan layer surrounding bacteria [11,12,13]. Many lectins display specific oligosaccharide-mediated bactericidal activity [14,15,16,17]. *O*-glycosylated oligosaccharides associated with major surface adhesive glycoproteins that contain *N*-acetyl d-galactosamine, *N*-acetyl d-glucosamine, d-rhamnose, or d-glucose have been found in various species of *Streptococcus* [18,19]. Lectins presumably affect the growth of SLO-producing *S. pyogenes*. We examined the effects of *Aplysia kurodai* lectin (AKL) on SLO hemolytic activity and on *S. pyogenes* growth.

## 2. Results and Discussion

### 2.1. Optimization of Hemolysis Assay by SLO

**Figure 1:**
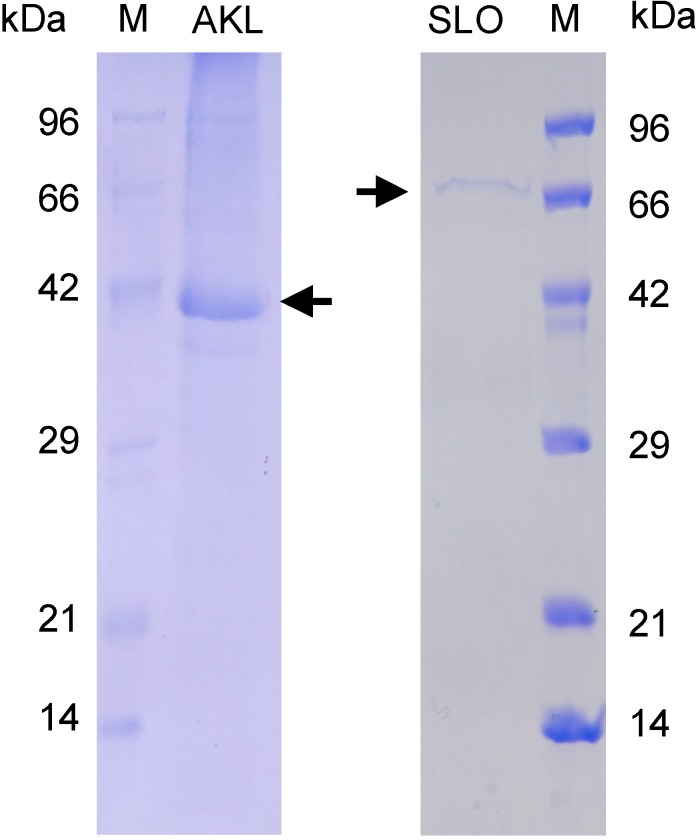
Purified AKL (**left**) and SLO (**right**) were subjected to SDS-PAGE under reducing condition. The standard marker proteins (M) used were phosphorylase b (96 kDa), bovine serum albumin (66 kDa), ovalbumin (42 kDa), carbonic anhydrase (29 kDa), trypsin inhibitor (21 kDa), and lysozyme (14 kDa).

**Figure 2:**
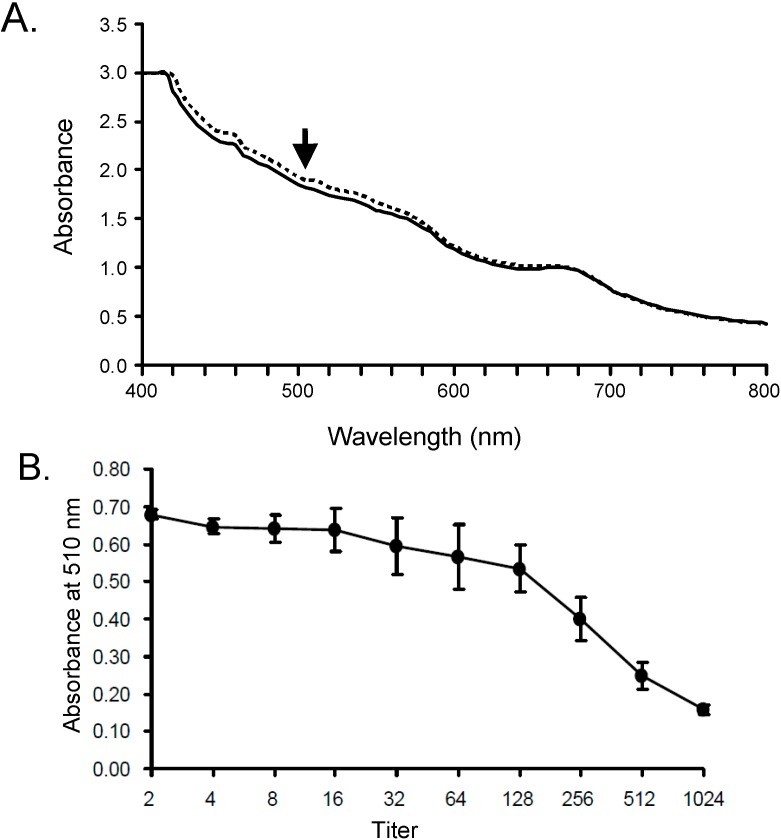
Stable absorbance of hemin chloride and optical detection of SLO-induced hemolysis. (**A**) 1 mL of RBCs ruptured by distilled water (dotted line), or RBCs hemolyzed by SLO (solid line), was mixed with 1 mL HCl (2%) in acetone to convert heme to hemin chloride. Arrow: optimal absorbance (wavelength 510 nm); (**B**) Serially 2-fold diluted SLO was mixed with ruptured RBCs and HCl (2%) in acetone, and absorbance at 510 nm was measured.

SLO was isolated from *S. pyogenes* culture medium, and found to have an Mr of ~64 kD (Figure 1, column SLO). SLO had a strong hemolytic effect on rabbit RBCs. To quantify hemolysis by OD analysis, equal volumes of HCl (2% v/v) in acetone and distilled water were added to the hemolyzed solution. This approach was quite effective; OD values for the two samples were nearly the same at the visible wavelength (510 nm) used for determination of hemolytic activity (Figure 2A). Comparative titration assay of the isolated exotoxin showed that OD value of 16-fold diluted SLO was similar to that of distilled water for RBC hemolysis (Figure 2B).

### 2.2. Specific Inhibition of SLO-Induced Hemolysis by AKL

**Figure 3:**
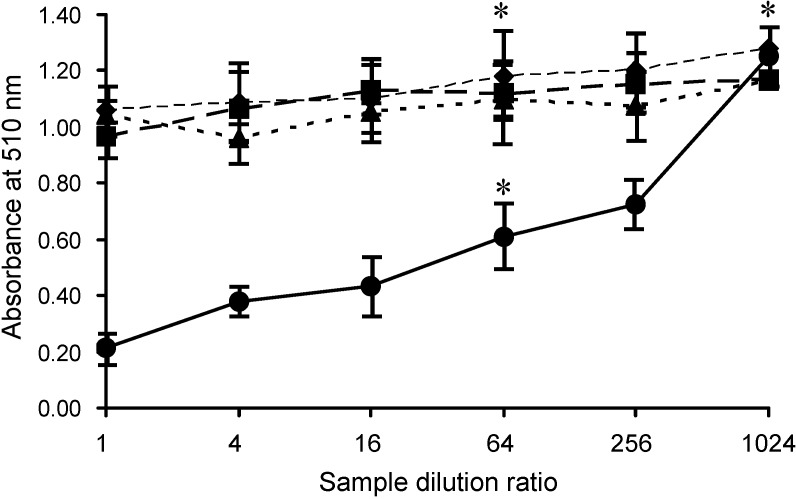
Effects of lectins on SLO-induced hemolysis. RBCs were pre-mixed with serially diluted lectins (AKL: solid line; ECA: dotted line; ABA: small dashed line; PNA: large dashed line). SLO was added to each sample, mixed with HCL/acetone, and inhibition of hemolysis was assessed by measurement of absorbance at 510 nm as in Figure 2. Error bars: SE from three independent experiments. * *p* < 0.01 in comparison with negative control.

**Figure 4:**
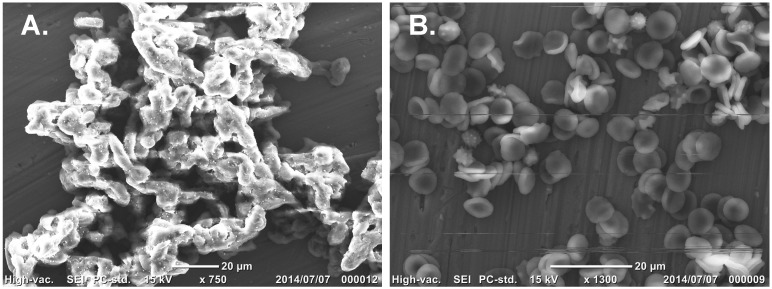
SEM imaging of RBCs agglutinated by AKL in the presence of SLO. (**A**) AKL-induced hemagglutination; (**B**) negative control (RBCs without AKL or SLO). Scale bars: 20 µm.

AKL was purified from sea hare eggs by melibiosyl-agarose column, and had Mr 40 kDa under reducing conditions (Figure 1, column AKL). The effects of AKL and of lectins from plants (ECA, PNA) and mushroom (ABA) on SLO-induced hemolysis were evaluated. Each of these lectins aggregated RBCs and did not cause hemolysis. AKL (but not the other lectins) had a dose-dependent inhibitory effect on SLO-induced hemolysis (Figure 3). This inhibitory effect was suppressed by the co-presence of 20 mM Gal, but not by 20 mM glucose (data not shown). AKL caused agglutination of RBC membranes, which were observed to be tightly fused by scanning electron microscopy (SEM) (Figure 4).

### 2.3. Treatment of RBCs with MβCD Blocks the Inhibitory Effect of AKL

**Figure 5:**
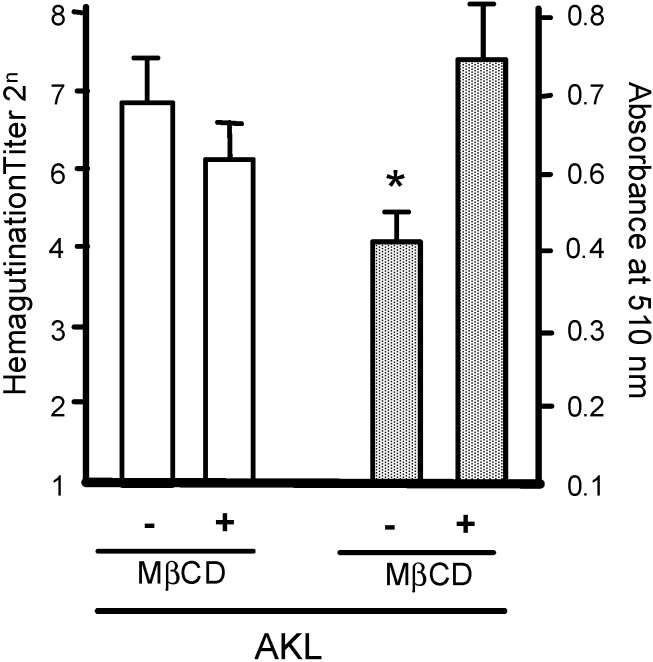
Pretreatment with MβCD suppresses the inhibitory effect of AKL on SLO-induced hemolysis. White columns: hemagglutination activity of AKL in RBCs pretreated with (+) or without (−) 12.5 mM of MβCD. Shaded columns: inhibitory effect of pretreatment with 12.5 mM of MβCD on hemolytic activity of 2 mg/mL AKL in RBCs in the presence of SLO. Error bars: SE from three independent cell preparations assayed individually. * *p* < 0.01 in comparison with negative control.

Treatment of RBCs with the cholesterol inhibitor MβCD significantly reduced the inhibitory effect of AKL on SLO-induced hemolysis (Figure 5). MβCD disrupts construction within the cell membrane of cholesterol-enriched microdomains, which contain many types of GSLs and glycoproteins that are recognized by AKL. These findings suggest that GSLs targeted by AKL are masked by MβCD, such that the lectin cannot bind to glycoconjugates located in cholesterol-enriched microdomains, and that MβCD has a dose-dependent inhibitory effect.

### 2.4. Inhibition of S. pyogenes Growth by AKL

**Figure 6:**
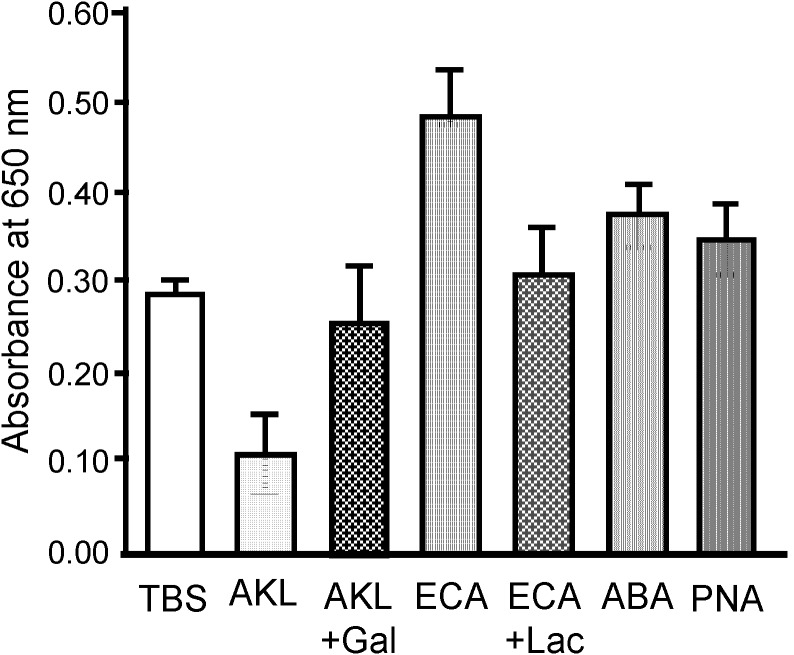
Effects of added lectins on growth of *S. pyogenes* in culture. *S. pyogenes* was grown in liquid culture with each of the indicated lectins (200 μg) for 18 h. The turbidity of the culture medium was assessed by measurement of absorbance at 650 nm. TBS: negative control, AKL: *Aplysia kurodai* galactose-binding lectin, AKL + Gal: AKL with 100 mM galactose, ECA: *Erythrina cristagalli* agglutinin, ECA + Lac: ECA with 100 mM lactose (Galâ1-4Glc), ABA: *Agaricus bisporus* agglutinin, PNA: peanut (*Arachis hypogaea*) agglutinin.

*S. pyogenes* growth as indicated by turbidity (OD value) was reduced in a dose-dependent manner by AKL (Figure 6). After 18 h incubation, OD value for bacteria treated with 2 mg/mL AKL was ~30% lower than for bacteria treated with the other lectins or no lectin (control). For 18 h incubation on a Muller Hinton II agar-coated plate containing 5% rabbit RBC, the numbers of live bacteria were respectively 4% and 39% less for AKL and ABA treatment than for control. In contrast, treatment with ECA, which recognizes type 2 lactosamine (Galβ1-4GlcNAc), caused a 180% increase of bacterial growth.

### 2.5. Stimulatory Effect of ECA on Growth of S. pyogenes

**Figure 7:**
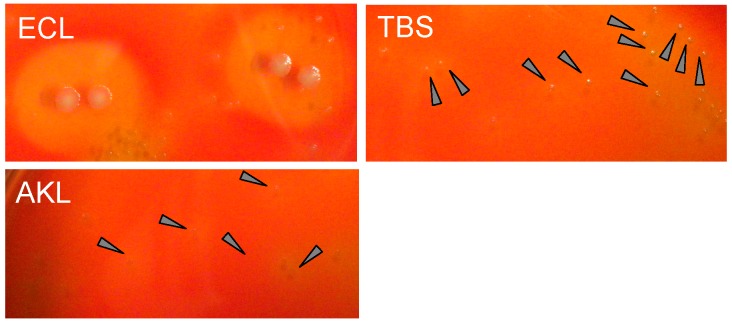
Stimulatory and inhibitory effects of ECA and AKL on growth of *S. pyogenes*. Culture media containing ECA, AKL, or TBS (as in Figure 6) were diluted 10-fold with saline. Each of the three media (100 μL) was spread on agar plates containing 5% rabbit RBCs and incubated for 18 h. Arrows indicate colonies.

The growth-stimulatory effect of ECA was evaluated by subculturing bacteria after incubation. Growth of *S. pyogenes* on agar plates was much higher for ECA treatment than for control (TBS only) whereas in presence of AKL, the growth of bacteria was minimal (Figure 7).

### 2.6. Bacteriostatic Activity of AKL

**Figure 8:**
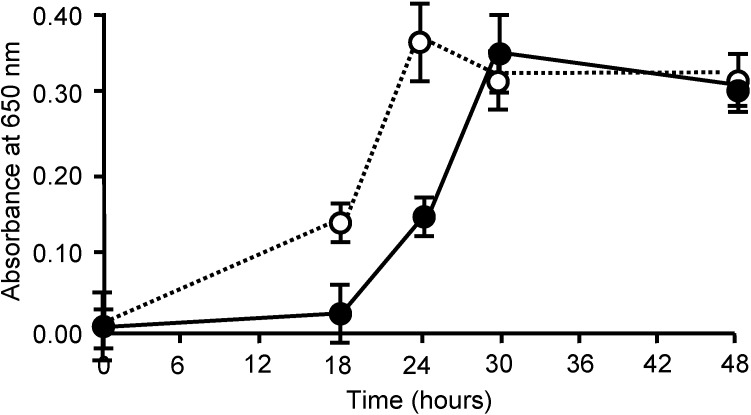
Delay of bacterial growth by addition of AKL. *S. pyogenes* cells (10^3^/mL) were incubated with (solid line) or without (dotted line) 2 mg/mL AKL. Growth was assessed based on turbidity (absorbance at 650 nm).

Bacteria cultured in the presence of AKL had growth rates lower than that of control at 18 and 24 h and took 6 h longer to reach maximal OD value (30 h, compared to 24 h for control) (Figure 8). The AKL-treated and -nontreated groups reached the same concentration after 48 h, indicating that AKL has a bacteriostatic but not bactericidal effect.

The 3-dimensional structure of SLO was elucidated recently, and the essential amino acids that bind cholesterol in the RBC membrane were identified by a structural biology approach [20,21]. These findings provide a basis for understanding the mechanism of pore formation and penetration of the RBC membrane by SLO in GEM through recognition of cholesterol.

We observed for the first time that SLO-induced hemolysis of RBCs is inhibited by pretreatment of RBCs with AKL, a d-Gal-binding lectin isolated from sea hare eggs. One possible mechanism is that lectin molecules are tightly stacked in the RBC membrane through GEM and inhibit SLO attachment, thereby blocking SLO-induced hemolysis. This concept was supported by SEM observations, which suggested that AKL prevented SLO from reaching the RBC membrane and thereby prevented hemolysis (Figure 4A).

We tested this possibility by treating RBCs with MβCD to eliminate the protective effect of AKL. MβCD has been shown to deplete cholesterol in GEM on the plasma membrane [22], inhibit lectin-mediated cell signaling [23,24], and block the induction of hemagglutination by Gal-binding lectins [25].

*A. kurodai* eggs have bright aposematic coloration and contain toxic molecules that deter potential predators. AKL, a Gal-binding lectin, may be one of these toxic molecules. We showed previously that AKL agglutinates rabbit and human RBCs without hemolysis, and has a cytotoxic effect on cultured mouse cells containing α-galactoside and complex-type oligosaccharides [9,10]. Rabbit and human RBCs contain GSLs with α-galactoside structures in membrane GEM [26]. This finding suggested that AKL might inhibit cholesterol-dependent, SLO-induced hemolysis.

For precise measurement of SLO-induced hemolysis and the inhibitory effect of AKL, chlorhemin generated by rupturing of RBCs by 50% (v/v) acetone containing 1% (v/v) HCl (final concentration) was convenient for detection. Absorbance of chlorhemin was stable at wavelengths from 400 to 800 nm, and was higher around 510 nm than that of hemoglobin (Figure 2A). SLO-induced hemolysis was inhibited by addition of AKL in this system (Figure 3). This finding suggests that the lectin causes tight stacking of cholesterol-containing GEM in RBC, thereby blocking penetration of the cell membrane by SLO. Consistent with this idea, Gal- and GalNAc-binding lectins (ECA, ABA, PNA) bound preferentially to oligosaccharides of glycoproteins but did not inhibit SLO-induced hemolysis, even though they agglutinated RBCs similarly to AKL. Clarifying the relationships among cholesterol, target molecules of SLO in GEM, and the stacking effect of AKL through carbohydrate-protein interaction, is the next step to understanding the inhibitory effect of AKL on SLO activity.

*Streptococcus* species have been reported to produce Gal and its derivatized polysaccharides such as Galβ1-3GalNAc and GalNAcβ1-3Gal [27]. *S. pyogenes* and other species are capable of producing biofilms whose growth is based on interactions between glycans [28]. Certain plant lectins independently modulate biofilm growth [29,30].

In the present study, AKL treatment for 18 h reduced bacterial growth by ~33%, whereas ECA (which recognizes *N*-acetyllactosamine [Galβ1-4GlcNAc]) promoted growth. Culture for 18 h with AKL or ABA reduced the proportion of living bacteria by 80% and 50%, respectively, in comparison with control. In contrast, ECA strongly increased the proportion of living cells (Figure 6), and bacterial growth (Figure 7). Growth of the bacteria was clearly affected in different ways by lectins with differing glycan-binding properties (Figure 8).

Our findings demonstrate the importance of glycoconjugates and glycan-binding receptors in regulating mechanisms of toxicity, creation of pores in the target cell membrane, and bacterial growth.

## 3. Experimental Section

### 3.1. Bacterial Strain, Culture, and Preparation of SLO

*Streptococcus pyogenes* (group A, type T-25) was obtained from the National Institute of Infectious Diseases, Tokyo, Japan. *S. pyogenes* were cultured and SLO was extracted as described previously [3,31]. Todd-Hewitt broth (3000 mL; Becton Dickinson; Franklin Lakes, NJ, USA) containing 1% yeast extract (Becton Dickinson) and 3% glucose was inoculated with 0.3 mL bacterial suspension. The culture was incubated at 37 °C with stirring for 12 h (pH 7.5), then centrifuged, concentrated to 100 mL with Aquacide II (Merck KGaA; Darmstadt, Germany), and used as crude SLO. The sample was stored at −30 °C with β-mercaptoethanol (2-ME; 1 mM) and used within 6 months.

### 3.2. Lectin Preparation

A Gal-binding *Aplysia kurodai* lectin (AKL) was purified from *A. kurodai* eggs as we described previously [9]. In brief, eggs (100 g) were homogenized in a mortar with 10 volumes (w/v) of 10 mM Tris (hydroxymethyl) aminomethane buffered saline (150 mM NaCl) adjusted to pH 7.5 with HCl (TBS). The homogenate was centrifuged at 27,500× *g* for 1 h at 4 °C, and the clear supernatant obtained was applied to a 5-mL melibiosyl-agarose column. The column was washed extensively with TBS, the lectin was eluted with 50 mM Gal in TBS, and 1-mL elution fractions were collected in tubes using a fraction collector. These fractions were combined and dialyzed against 1,000 volumes of TBS to remove Gal. Three other Gal-binding lectins derived from coral tree seeds (*Erythrina cristagalli*; ECA), mushroom fruiting bodies (*Agaricus bisporus*; ABA), and peanuts (*Arachis hypogaea*; PNA) were obtained from Cosmo Bio Co. (Tokyo, Japan). Protein concentrations were determined with a BCA protein assay kit [32,33] and absorbance measured at wavelength 562 nm. Lectin Mr values were determined by SDS-PAGE at a constant current of 30 mA for 1 h [34].

### 3.3. RBC Preparation

Fresh rabbit blood (3 mL) was collected from the ear vein with a 21-gauge needle and transferred to a plastic tube with 3.8% (w/v) sodium citrate in saline in TBS (300 μL). All procedures were performed according to the guidelines outlined in the institutional Animal Care and Use Committee of the Yokohama City University, Yokohama Japna. RBCs were washed three times with 10× volume of TBS by centrifugation (2500 rpm) for 15 min at room temperature. A 2.5% (v/v) RBC suspension was prepared by diluting with TBS (2 mL RBC suspension was placed in a tube and the volume increased to 10 mL with TBS). To maintain a constant 2.5% RBC concentration in all analyses, 500 μL RBC suspension was ruptured in 9.5 mL distilled water, and absorbance was measured. An absorbance value of 0.28 ± 0.005 at 560 nm indicated a stable concentration of RBC suspension.

### 3.4. Hemagglutination Assay and Sugar Binding Specificity

Hemagglutination assay was performed in 96-well V-shaped plates as described previously [35]. Twenty μL of 2-fold diluted purified lectin in TBS was mixed with 20 μL of a 1% suspension (with TBS; v/v) of rabbit RBCs in 20 μL TBS. The plate was incubated at room temperature for 1 h. Formation of a sheet (agglutination-positive) or dot (agglutination-negative) was observed and scored as hemagglutination titer.

### 3.5. Hemolytic Activity of SLO

Hemolysis assays were performed in 4-mL polypropylene tubes. SLO (250 μL) was serially 2-fold diluted with TBS containing 1% bovine serum albumin (BSA). TBS (250 μL) containing 2-ME (1 mM), 2.5% (v/v) rabbit RBCs in TBS (0.5 mL), and TBS containing 1% BSA (1 mL) were added sequentially to each tube. Tubes were incubated at 37 °C for 60 min, centrifuged at 2500 rpm for 15 min, and added with 2% HCl in acetone (1 mL per 1 mL sample solution) to convert heme to hemin chloride. Optimal visible absorbance was evaluated using a spectrophotometer (model Genesis 10 Vis/UV, Thermo Fisher Scientific; Waltham, MA, USA) [36].

### 3.6. SEM Observationof AKL-Induced Hemagglutination in the Presence of SLO

Rabbit RBCs were washed three times with Tris-buffered saline (TBS: 10 mM Tris(hydroxymethyl)aminomethane (pH 7.4), 150 mM NaCl) by centrifugation at 1000× *g* for 10 min at 4 °C. An RBC sample (1 mL; 1% v/v) was washed with 1% BSA and 2-ME (1 mM) in the presence or absence of AKL and SLO (10 µg/mL each), fixed with 1% glutaraldehyde for 1 h at room temperature, washed in TBS, and immersed for 30 min each in a *t*-butanol/ethanol mixture (1:1 v/v) and absolute *t*-butanol. The sample was dried, sputtered with gold in a magnetron sputter (model MSP-mini; JEOL Ltd.; Tokyo, Japan), and observed by SEM (model JCM6000; JEOL Ltd.; Tokyo, Japan).

### 3.7. Evaluation of AKL-Dependent Inhibition of Hemolysis Using Rbcs Treated with Methyl-β-d-Cyclodextrin

RBCs (2 × 10^5^) prepared as above were mixed with fresh medium with or without 12.5 mM methyl-β-d-cyclodextrin (MβCD) (Wako Pure Chemical Industries; Osaka, Japan). Cell surface cholesterol in GEM was almost depleted by treatment with MβCD for 1 h [37]. To evaluate the possible effect of GEM on AKL-induced hemagglutination, the hemagglutination assay described above was performed with addition of serially diluted AKL (original titer adjusted to 120).

### 3.8. Effects of Lectins on SLO-Induced Hemolysis

Four lectins (AKL, ECA, ABA, PNA) were dissolved separately in TBS containing 1% BSA, and 4 mg/mL solutions were prepared. The solutions (250 μL) were serially 2-fold diluted with TBS containing 1% BSA. Each tube was added with 250 μL SLO (16-fold diluted with TBS) containing 2-ME (1 mM), incubated with 2.5% rabbit RBCs in TBS (500 μL) or 1% BSA containing TBS (1 mL) at 37 °C for 60 min, and centrifuged at 2500 rpm for 15 min. One mL sample solution was mixed with 1 mL of 2% HCl in acetone. A 200-μL aliquot of each sample was transferred into a plastic 96-well flat-bottom microplate, and absorbance at 510 nm was measured using a plate reader (Epoch Microphotometer; BioTek; Winooski, VT, USA).

### 3.9. Effects of Lectins on Growth of S. pyogenes

*S. pyogenes* cells were cultured in brain heart infusion broth (Becton Dickinson) for 24 h at 37 °C and adjusted to a concentration at 10^3^/mL by dilution with Todd Hewitt broth containing 0.1% yeast extract. Aliquots (100 μL) of these diluted cells were cultured with various concentrations of lectins in 96-well microplates for 18 h at 37 °C as described previously [3]. Sufficient bacterial growth was assessed based on turbidity in the medium (optical density [OD] 1.0 at 650 nm). Bacteria were subcultured after incubation to evaluate the effects of lectins on cell growth. The medium was diluted 10-fold by saline, and 100 μL of the resulting solution was spread on Muller Hinton II agar (15-mL plate; Becton Dickinson) containing 5% (v/v) rabbit RBCs for 18 h at 37 °C. Numbers of living colonies were counted, and bacterial growth rates were monitored followed lectin addition.

### 3.10. Statistical Analysis

Experimental data are presented as mean ± standard error (SE). Differences between means were evaluated by two-tailed Student’s *t*-test, with *p* values <0.05 considered to be statistically significant.

## 4. Conclusions

AKL, a Gal-binding lectin isolated from *Aplysia kurodai* eggs, specifically inhibited SLO-induced hemolysis, although SLO was not affected by glycans. SEM observations indicated that AKL tightened the junctions between RBC membranes through recognition of glycans in GEM, suggesting that SLO was unable to induce hemolysis because it was prevented from penetrating the spaces between membranes. AKL also caused a >6-h delay in growth of *Streptococcus pyogenes* in comparison with control.
