# Correction: Hasan, I., *et al*. A Galactose-Binding Lectin Isolated from *Aplysia kurodai* (Sea Hare) Eggs Inhibits Streptolysin-Induced Hemolysis. *Molecules* 2014, *19*, 13990–14003

**DOI:** 10.3390/molecules21010129

**Published:** 2016-01-21

**Authors:** Imtiaj Hasan, Miharu Watanabe, Naoto Ishizaki, Yoshiko Sugita-Konishi, Yasushi Kawakami, Jun Suzuki, Chikaku Dogasaki, Sultana Rajia, Sarkar M. A. Kawsar, Yasuhiro Koide, Robert A. Kanaly, Shigeki Sugawara, Masahiro Hosono, Yukiko Ogawa, Yuki Fujii, Hideyuki Iriko, Jiharu Hamako, Taei Matsui, Yasuhiro Ozeki

**Affiliations:** 1 Laboratories of Glycobiology & Marine Biochemistry and Molecular Toxicology, Department of Life and Environmental System Science, Graduate School of NanoBio Sciences, Yokohama City University, 22-2 Seto, Kanazawa-ku, Yokohama 236-0027, Japan; hasanimtiaj@yahoo.co.uk (I.H.); rajia_bio@yahoo.com (S.R.); yasukoide04@yahoo.co.jp (Y.K.); kanaly@yokohama-cu.ac.jp (R.A.K.); 2 School of Life and Environmental Science, Azabu University, 1-17-71, Fuchinobe, Chuo-ku, Sagamihara, Kanagawa 252-5201, Japan; salty-soybean@kuh.biglobe.ne.jp (M.W.); ishizaki@azabu-u.ac.jp (N.I.); y-konishi@azabu-u.ac.jp (Y.S-K.); yasushi@azabu-u.ac.jp (Y.K.); suzukij@azabu-u.ac.jp (J.S.); dogasaki@azabu-u.ac.jp (C.D.); 3 Department of Biochemistry and Molecular Biology, Faculty of Science, University of Rajshahi, Rajshahi-6205, Bangladesh; 4 Department of Natural Science, Varendra University, Rajshahi-6204, Bangladesh; 5 Department of Chemistry, Faculty of Sciences, University of Chittagong, Chittagong-4331, Bangladesh; akawsarabe@yahoo.com; 6 Division of Cell Recognition Study, Institute of Molecular Biomembrane and Glycobiology, Tohoku Pharmaceutical University, 4-4-1 Komatsushima, Aoba-ku, Sendai 981-8558, Japan; ssuga@tohoku-pharm.ac.jp (S.S.); mhosono@tohoku-pharm.ac.jp (M.H.); 7 Department of Pharmacy, Faculty of Pharmaceutical Science, Nagasaki International University, 2825-7 Huis Ten Bosch, Sasebo, Nagasaki 859-3298, Japan; yogawa@niu.ac.jp (Y.O.); yfujii@niu.ac.jp (Y.F.); 8 Department of Parasitology, Graduate School of Health Sciences, Kobe University, 7-10-2, Tomogaoka, Suma-ku, Kobe 654-0142, Japan; iriko@koala.kobe-u.ac.jp; 9 Department of Biology, School of Health Sciences, Fujita Health University, Toyoake, Aichi 470-1192, Japan; jhamako@fujita-hu.ac.jp (J.H.); tmatsui@fujita-hu.ac.jp (T.M.)

The authors wish to make the following correction to their paper [1]. In the Experimental Section (3.3. RBC Preparation) on page 13998, “Fresh rabbit blood (3 mL) was collected from the ear vein with a 21-gauge needle and transferred to a plastic tube with 3.8% (*w*/*v*) sodium citrate in saline in TBS (300 μL). All procedures were performed according to the guidelines outlined in the institutional Animal Care and Use Committee of the Yokohama City University, Yokohama Japan” should be replaced with “Rabbit erythrocyte cells were purchased from a commercial supplier (Kohjin Bio Co., Ltd., Sakado, Saitama, Japan) and used following the standard procedure.” The conclusions of the article remain unchanged.

The authors would like to apologize for any inconvenience caused to the readers by this change.
